# Utilization of dental services and associated factors among preschool children in China

**DOI:** 10.1186/s12903-019-0996-x

**Published:** 2020-01-08

**Authors:** Xiaoli Gao, Min Ding, Mengru Xu, Huijing Wu, Chunzi Zhang, Xing Wang, Xiping Feng, Baojun Tai, Deyu Hu, Huancai Lin, Bo Wang, Shuguo Zheng, Xuenan Liu, Wensheng Rong, Weijian Wang, Chunxiao Wang, Yan Si

**Affiliations:** 1 Department of Preventive Dentistry, Peking University School and Hospital of Stomatology, National Engineering Laboratory for Digital and Material Technology of Stomatology, Beijing Key Laboratory of Digital Stomatology, 22 Zhongguancun South Avenue, Haidian District, Beijing, 100081 China; 2 Peking University School and Hospital of Stomatology, National Engineering Laboratory for Digital and Material Technology of Stomatology, Beijing Key Laboratory of Digital Stomatology, Chinese Stomatological Association, Beijing, China; 3 Department of Preventive Dentistry, Shanghai Ninth People’s Hospital, Shanghai Jiao Tong University School of Medicine, Shanghai, China; 4 Department of Preventive Dentistry, State Key Laboratory Breeding Base of Basic Science of Stomatology (Hubei-MOST) and Key Laboratory of Oral Biomedicine Ministry of Education, School and Hospital of Stomatology, Wuhan University, Wuhan, China; 5 Department of Preventive Dentistry, West China School of Stomatology, Sichuan University, Chengdu, China; 6 Department of Preventive Dentistry, Guanghua School of Stomatology, Hospital of Stomatology, Sun Yat-sen University and Guangdong Provincial Key Laboratory of Stomatology, Guangzhou, China; 7 Center for Chronic and Non-communicable Disease Control and Prevention, Chinese Center for Disease Control and Prevention, No. 27 Nanwei road, Xicheng district, Beijing, 100050 China

**Keywords:** Utilization of dental services, China, Preschool children

## Abstract

**Background:**

This study sought to evaluate dental utilization among 3-,4-, and 5-year-old children in China and to use Andersen’s behavioural model to explore influencing factors, thereby providing a reference for future policy making.

**Methods:**

This study is a cross-sectional study. Data of 40,305 children aged 3–5 years were extracted from the Fourth National Oral Health Survey, which was performed from August 2015 to December 2016. Patient data were collected using a questionnaire, which was answered by the child’s parents, and clinical data were collected during a clinical examination. Stratification and survey weighting were incorporated into the complex survey design. Descriptive statistics, bivariate correlations and hierarchical logistic regression results were then analysed to find the factors associated with oral health service utilization.

**Results:**

The oral health service utilization prevalence during the prior 12 months were 9.5% (95%CI: 8.1–11.1%) among 3-year-old children, 12.1% (95%CI: 10.8–13.5%) among 4-year-old children, and 17.5% (95%CI: 15.6–19.4%) among 5-year-old children. “No dental diseases” (71.3%) and “dental disease was not severe” (12.4%) were the principal reasons why children had not attended a dental visit in the past 12 months. The children whose parents had a bachelor’s degree or higher (OR: 2.29, 95%CI: 1.97–2.67, *p* < 0.001), a better oral health attitude ranging from 5 to 8(OR: 1.64, 95%CI: 1.43–1.89, *p* < 0.001), annual per capital income more than 25,000 CNY (OR: 1.40, 95%CI: 1.18–1.65, *p* < 0.001),think their child have worse or bad oral health (OR: 3.54, 95%CI: 2.84–4.40, *p* < 0.001), and children who often have toothaches (OR: 9.72, 95%CI: 7.81–12.09, *p* < 0.001) were more likely to go to the dentist in the past year.

**Conclusion:**

The prevalence of dental service utilization was relatively low among preschool children. It is necessary to strengthen oral health education for parents and children, thereby improving oral health knowledge as well as attitude, and promoting dental utilization.

## Background

Early childhood caries (ECC) is a widespread public health concern and is defined as the presence of one or more decayed (non-cavitated or cavitated lesions), missing (due to caries) or filled tooth surfaces (dmfs) in any primary (deciduous) tooth in a child aged 6 years or younger [1]. In China, the prevalence of ECC at age 5 ranged from 66.0% in the Third National Oral Health Epidemiological Survey to 71.9% in the Fourth National Oral Health Epidemiological Survey [2, 3]. ECC prevalence showed a substantial increasing trend over time, it still remains at a relatively high level. Hence, dental care needs continue to be unmet in our society.

Previous research has found that the mean number of decayed, missing and filled teeth (dmft) among children who had been to a dentist within the previous 12 months was much lower than that in those who had never visited a dentist [4]. It can be concluded that regular check-ups play an important role in preventing the development of ECC. Regular check-ups could diminish treatment costs, ensure a child’s healthy growth, and improve oral health-related quality of life [1]. Another study suggested that 94% of the sample population reported attending regular dental visits after the first visit [5]. The American Academy of Pediatric Dentistry (AAPD) recommends that children attend their first dentist visit upon eruption of the first tooth or no later than 1 year of age [6].

There is no doubt that parental behaviour has an important influence on the good oral health care habits of children. However, a large portion of the general public mistakenly feels that children do not need to see a dentist during the period of primary dentition [7]. In one study in a Hong Kong population, only 44% of parents sought treatment even though free dental care was offered to their children [8]. Furthermore, oral health service utilization varies from country to country. In a developed country, 41.9% of American children reported an annual dental visit for general dental care [9]. Moreover, in Belgium, 38 and 79% of children had utilized dental services at the ages of 3 and 5 years, respectively [10]. For developing country, Brazil, Baldani, et al. find that the pattern of lack of access to dental care has remained unchanged for pre-schoolers over the years [11]. Based on the Third National Oral Health Epidemiological Survey, only 15% of 5-year-old children have utilized dental services [2]. Therefore, understanding dental visit patterns is important to diminish disparities and inequalities.

Several studies [12] have focused on identifying factors associated with oral health service utilization via theoretical frameworks; one of the most well-known frameworks is Andersen’s model. Created in the late 1960s, Andersen’s model has become a dynamic and recursive health service model that has been developed in 4 phases [13]. In the initial behaviour model, it was suggested that people’s use of health services was dependent on the following: (1) predisposing factors that existed prior to the development of a specific illness, indicating a propensity towards use according to individual characteristics (e.g., age, sex, education); (2) enabling factors that determine the health service resources available and accessible to the individual (e.g., income, insurance coverage); and (3) the need for care, including both self-perceived and clinically evaluated needs. The revised model also recognized that personal health practices, such as diet, exercise, and self-care, interacted with the utilization of health services to influence health outcomes [13, 14].

Prior studies using Andersen’s behavioural model have shown that oral health services may be influenced by parental education levels, perception of the child’s quality, dental insurance and pain [15–17]. Additionally, characteristics such as poor oral health knowledge and poor oral health attitudes are frequently cited as barriers to dental care [18, 19]. In China, there are few studies on the utilization of oral health services, especially studies involving children. Xu et al. assessed oral health service utilization patterns among preschool children in Beijing, China, and found strong associations between dental pain, available oral health care resources and dental service utilization [20]. In addition, In Chongqing, Wang et al. found that the point of brushing teeth every day and a history of toothache in the past year can also affect the prevalence of oral visits [21]. However, the existing studies have narrowly focused on local areas, and there are few detailed and systematic studies on the influencing factors of oral health care utilization, resulting in crosswise comparisons.

This study was part of the Fourth National Oral Health Epidemiological Survey and aimed to evaluate dental utilization among 3-,4-, and 5-year-old children in China. Andersen’s behavioural model was used to investigate influencing factors, thereby providing a reference for future policy making.

## Methods

The Fourth National Oral Health Survey was carried out from August 2015 to December 2016. Data for the 3- to 5-year-old age groups were extracted; the specific study methodology has been detailed in a previous study [22]. A multistage cluster, random, equal proportion sampling method was adopted for this survey.

All 31 provinces, autonomous regions and municipalities, including Tibet in mainland China, were included. Then, the population was stratified into urban and rural residents. We selected two urban and two rural districts randomly from each province using probability proportional to size (PPS) sampling. Next, we used the PPS method to randomly select three streets in urban districts and three townships in rural districts. Last, from each district, three kindergartens were selected by PPS sampling; After their guardians signed the informed consent form, a total of 40,305 children from 372 kindergartens were included in the final analysis. The data collected in this study mainly included an oral health examination and questionnaire survey (see Additional files 1 and 2). Inclusion criteria: 1) Age 3–5 years; 2) Resident time in the local area > 6 months; 3) Informed consent of parents. Exclusion criteria: Not meeting the above conditions.

The clinical data were collected by clinical examination according to the World Health Organization (WHO) methodology and criteria. All participants identified in the survey were examined by trained dentists using disposable dental mirrors and community periodontal index (CPI) probes under artificial light. To control for quality, standard assessments of consistency were conducted throughout the survey. All examiners were trained by a standard examiner. All qualified examiners were required to meet the condition that the inter-examiner kappa value was greater than 0.8 for the dmft index. During the survey, duplicate examinations were performed for 5% of the examinations to ensure consistency, and the kappa value reached 0.95 in the 3–5-year-old group.

A questionnaire was sent to the children’s parents. The questionnaire included questions on the children’s background information (i.e., age, sex, urban or rural, region), demographic information (i.e., household income, number of family members, parental education level), oral health knowledge, the attitude of parents, dental pain in the children, the parental evaluation of the health status of their children, and dental attendance experience. The questionnaire was answered by the child’s parents, mainly the caregivers in their daily life.

To facilitate statistical analysis, we processed the questions. Data regarding attitude and knowledge were measured by a scoring system. The respondent scored 1 point if he or she showed a positive attitude. When the scores of all eight questions were summed, the total attitude score ranged from 0 to 8. For the analysis, we regarded a score of 0–4 points as a negative attitude and 5–8 points as a positive attitude. Similar to the above method, the respondent received a total score for knowledge-related questions (one point for each correct answer and a total of six questions). The scores from responses to these questions were classified as indicating poor (0–3 points) or good (4–6 points) oral health knowledge. The annual per capita household income was calculated by dividing the total annual household income by the number of households. However, 1362 data points for the number of households and 14,982 data points for the total annual household income were missing. To estimate the parameter more accurately and make full use of the existing data, the expectation-maximization (EM) algorithm was applied to account for the missing values.

The statistical analyses were performed using STATA 14 (Stata Corporation, College Station, TX). Although this survey encompassed a complex sampling, sampling weights were disregarded during the investigation. To reduce sampling error, the samples were post-stratified according to sex, province and urban-rural classification. Survey weights were then computed by comparing the population of each stratum in the sample with the population of that stratum determined in the Sixth Population Census of China [23]. Descriptive analyses were performed to characterize each study variable. Afterwards, chi-square tests and t-tests were used to analyse the association between the independent variables and outcome variables. Statistical significance was set at 0.05. A hierarchical logistic regression was performed for all variables with significance in the bivariate correlation analyses, and statistical significance was set at 0.05. All reported *p* values are two-tailed.

## Results

### Descriptive characteristics of the participants

In the 3- to 5-year-old age group, our final analytic sample consisted of 40,305 participants, among which 17.6% (95%CI:16.1–19.3%) of the children were reported to have utilized oral health services, and 13.1% (95%CI:11.8–14.5%) of the children had visited a dentist in the past 12 months. The oral health service utilization in each subgroup was 9.5% (95%CI: 8.1–11.1%) among 3-year-old children, 12.1% (95%CI: 10.8–13.5%) among 4-year-old children, and 17.5% (95%CI: 15.6–19.4%) among 5-year-old children in the past 12 months.

Table 1 presents a summary of the descriptive characteristics of the participants. The participants were 50.2% male. The participants were from east (35.0%), west (39.3%) and central (25.8%) China. Participants from urban and rural areas were approximately equal. Most parents reported positive oral health attitudes (87.7%) and high levels of knowledge (63.8%). A total of 68.7% of parents had a high school education or less, while 31.3% of parents received a bachelor’s degree or higher. Nearly 30.4% of the participants had an annual per capita income of less than 12,500 CNY., 36.9% of the participants had an annual per capita income between 12,500 CNY and 25,000 CNY, and 32.7% of the participants had an annual per capita income of more than 25,000 CNY. Only 2.5% of the parents perceived their children’s overall health status as “poor”; however, 10.6% of parents evaluated their children’s oral health status as “poor”. Most children (70.0%) reported that they never experienced dental pain. In this study, the prevalence of caries was 63.1%.

**Table 1:** Descriptive characteristics of the sample of preschool children aged 3–5 years

	% (95%CI)		% (95%CI)
Predisposing factor			
Age group		**Annual per capital income**	
3	30.7% (30.3–31.2%)	the lowest to 12,500CNY	30.4% (30.0–30.9%)
4	34.6% (34.2–35.1%)	12,5000CNY-25,000CNY	36.9% (36.4–37.3%)
5	34.7% (34.2–35.1%)	25,000CNY to the highest	32.7% (32.3–33.2%)
Sex		**Need factor**	
male	50.2% (49.7–50.7%)	**Toothache**	
female	49.8% (49.3–50.3%)	never	70.0% (69.6–70.5%)
Education			
junior high school or lower	45.3% (44.8–45.8%)	occasionally	23.2% (22.8–23.6%)
senior high school	23.4% (23.0–23.8%)	often	2.6% (2.4–2.7%)
bachelor’s degree or higher	31.3% (30.8–31.7%)	It’s not known	4.2% (4.0–4.4%)
Score of oral health attitude		**Evaluation of overall health**	
0–3	12.3% (12.0–12.6%)	good	30.3% (29.8–30.7%)
4–6	87.7% (87.4–88.0%)	fair	41.3% (40.8–41.8%)
Score of oral health knowledge		moderate	26.0% (25.5–26.4%)
0 to 4	36.2% (35.7–36.6%)	worse or bad	2.5% (2.3–2.6%)
5 to 8	63.8% (63.4–64.3%)	**Evaluation of oral health**	
Enabling factor		good	20.5% (20.2–20.9%)
Location		fair	35.3% (34.8–35.7%)
urban	50.7% (50.2–51.2%)	moderate	33.6% (33.2–34.1%)
rural	49.3% (48.8–49.8%)	worse or bad	10.6% (10.3–10.9%)
Region		**dmft**	
west	39.3% (38.8–39.7%)	< 0	36.9% (34.3–39.7%)
middle	25.8% (25.4–26.2%)	> 0	63.1% (60.3–65.7%)
east	35.0% (34.5–35.4%)		

*CNY* Chines Yuan

*dmft* decayed, missing and filled teeth

### Reasons and barriers for visiting a dentist in the past 12 months

**Fig. 1:**
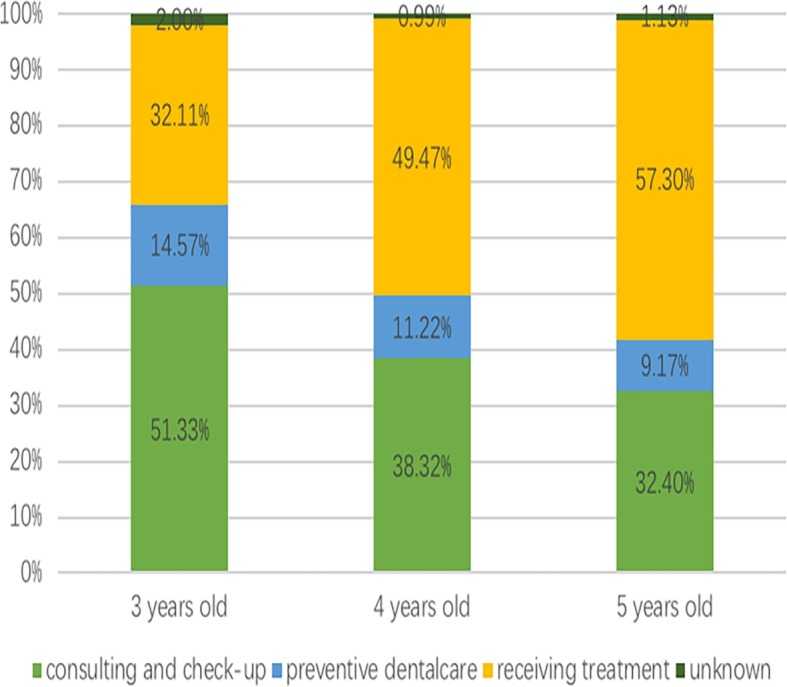
The percentage of the reasons for the last dental visit in the past 12 months

The reasons for the last dental visit among those who visited a dentist in the past 12 months are shown in Fig. 1. In summary, 32.11% of children aged 3, 49.47% of children aged 4, and 57.30% of children aged 5 visited a dentist for treatment, 14.57% of children aged 3, 11.22% of children aged 4, and 9.17% of children aged 5 visited a dentist for preventive dentalcare, and most of the other children visited only for consultations and check-ups. It can be seen that as age increases, the proportion of “preventive dental care” decreases, whereas the proportion of “receive treatment” increases.

Table 2 shows the barriers to dental visits in the past 12 months. Most participants in the three groups reported not visiting a dentist because of “no dental diseases”. Approximately 10.0% of participants cited “dental disease was not severe” as their reason for not obtaining dental care. Only less than 1% of children did not visit a dentist because of “difficulty of registration”.

**Table 2:** Reasons for not visiting a dentist in the past 12 months

Reasons	3 year old(%)	4 year old(%)	5 year old(%)	3–5 year old(%)
No dental diseases	76.80%	71.00%	66.10%	71.30%
Dental disease was not severe	10.00%	12.40%	14.80%	12.40%
No need to cure primary teeth	8.00%	8.90%	12.90%	9.90%
Economic issue	1.10%	1.10%	1.30%	1.20%
Inconvenience	1.80%	1.90%	2.00%	1.90%
No time	4.90%	5.50%	5.80%	5.40%
Fear of pain	3.40%	4.10%	4.20%	3.90%
No dentists nearby	1.60%	1.90%	1.80%	1.80%
Fear of infectious diseases	0.90%	1.50%	0.90%	1.10%
No reliable dentists	2.10%	2.50%	2.30%	2.30%
Difficulty of registration	0.50%	0.50%	0.60%	0.50%
Seeing dentists in kindergarten	3.00%	4.00%	4.70%	3.90%
Other reasons	7.10%	7.20%	7.00%	7.10%

### Factors associated with oral health service utilization in the past 12 months

Bivariate correlations between dental care utilization and the study variables are summarized in Table 3. We examined the factors influencing the use of dental health services among the three age groups. The predisposing factor variables, such as education, knowledge, and attitude were significantly related to the children’s utilization of oral health services. The enabling variables, including annual per capita income and rural-urban locations, had a significant association with the frequency of dental care. The need variables, such as dental pain, evaluated dental health, and dmft scores (mean:3.42, SD:4.220) were also significantly associated with dental visits, reflecting dental need.

**Table 3:** Bivariate comparisons of oral health service utilization in the past 12 months

Predisposing factor	3 years old		4 years old		5 years old		3–5 years old	
	% (95%CI)	P	% (95%CI)	P	% (95%CI)	P	% (95%CI)	P
Sex		=0.197		=0.432		=0.207		=0.696
male	9.9% (8.2–11.9%)		11.9% (10.4–13.5%)		17.1% (15.3–19.0%)		13.0% (11.7–14.6%)	
female	9.0% (7.7–10.5%)		12.4% (11.1–13.8%)		17.9% (16.0–20.1%)		13.2% (11.9–14.6%)	
Education		< 0.001		< 0.001		< 0.001		< 0.001
junior high school or lower	5.3% (4.2–6.6%)		8.8% (7.6–10.2%)		12.3% (10.7–14.0%)		9.0% (8.0–10.1%)	
senior high school	9.5% (8.0–11.1%)		12.2% (10.6–13.9%)		18.4% (16.2–20.9%)		13.5% (12.1–15.0%)	
bachelor’s degree or higher	16.4% (14.3–18.9%)		18.8% (16.3–21.6%)		27.9% (25.3–30.8%)		20.9% (19.0–22.9%)	
Score of oral health attitude		< 0.001		< 0.001		< 0.001		< 0.001
0 to 3	3.7% (2.7–5.1%)		5.5% (4.4–7.0%)		9.5% (7.9–11.4%)		6.5% (5.5–7.6%)	
4 to 6	10.3% (8.8–12.0%)		13.0% (11.6–14.5%)		18.7% (16.9–20.7%)		14.1% (12.8–15.5%)	
Score of oral health knowledge		< 0.001		< 0.001		*P* < 0.001		*P* < 0.001
0 to 4	6.1% (5.2–7.2%)		8.7% (7.4–10.3%)		12.3% (10.8–13.9%)		9.2% (8.3–10.3%)	
4 to 8	11.3% (9.5–13.4%)		14.1% (12.6–15.7%)		20.8% (18.8–23.0%)		15.4% (13.9–17.1%)	
Enabling factor								
Location		< 0.000		< 0.000		< 0.000		< 0.000
urban	13.1% (10.8–15.9%)		15.4% (13.1–18.1%)		22.7% (19.8–25.9%)		17.2% (14.9–19.8%)	
rural	6.9% (5.4–8.9%)		9.8% (8.4–11.4%)		13.7% (11.8–15.8%)		10.2% (8.9–11.7%)	
Region		=0.283		=0.023		=0.003		=0.011
east	9.3% (6.7–12.8%)		10.2% (8.4–12.0%)		14.5% (12.1–17.3%)		11.4% (9.4–13.8%)	
middle	8.1% (6.2–10.5%)		11.1% (8.8–13.8%)		15.5% (12.6–18.8%)		11.6% (9.7–13.8%)	
west	11.0% (8.5–14.2%)		14.5% (12.1–17.3%)		21.5% (18.1–25.3%)		15.9% (13.3–18.8%)	
Annual per capital income		< 0.001		< 0.001		< 0.001		< 0.001
the lowest to 12,500CNY	6.0% (4.5–7.8%)		8.8% (7.1–10.8%)		13.1% (11.2–15.3%)		9.4% (8.1–11.0%)	
12,5000CNY-25,000CNY	10.4% (8.7–12.3%)		12.7% (11.4–14.2%)		17.5% (15.5–19.6%)		13.6% (12.2–15.1%)	
25,000CNY to the highest	12.8% (11.1–14.7%)		15.7% (13.4–18.2%)		23.3% (20.5–26.3%)		17.4% (15.6–19.3%)	
Need factor								
Toothache		< 0.001		< 0.001		< 0.001		< 0.001
never	6.0% (4.8–7.5%)		6.0% (5.0–7.1%)		7.2% (6.1–8.4%)		6.3% (5.4–7.4%)	
occasionally	26.9% (23.3–30.9%)		29.5% (26.4–32.8%)		33.7% (30.4–37.2%)		30.9% (28.4–33.6%)	
often	37.7% (30.4–45.6%)		50.4% (43.2–57.5%)		58.5% (51.7–65.0%)		52.7% (47.6–57.7%)	
its not known	5.1% (3.3–8.0%)		6.9% (4.7–10.2%)		10.9% (75.8–15.5%)		7.5% (5.7–9.9%)	
Evaluation of overall health		=0.004		=0.692		=0.038		=0.030
good	9.6% (7.8–11.8%)		12.1% (10.4–14.0%)		17.0% (15.0–19.3%)		13.1% (11.6–14.7%)	
fair	10.2% (8.7–11.9%)		12.6% (10.9–14.5%)		19.0% (16.6–21.7%)		13.9% (12.4–15.6%)	
moderate	8.7% (7.0–10.7%)		11.6% (10.1–13.2%)		15.6% (13.5–18.0%)		12.1% (10.8–13.5%)	
worse or bad	4.9% (2.7–8.7%)		10.8% (7.1–16.3%)		19.5% (14.1–26.4%)		12.2% (9.5–15.6%)	
Evaluation of oral health		< 0.001		< 0.001		< 0.001		< 0.001
good	5.3% (4.0–7.0%)		4.5% (3.5–5.8%)		6.6% (5.1–8.4%)		5.4% (4.5–6.6%)	
fair	6.7% (5.4–8.4%)		8.1% (6.9–9.5%)		10.3% (8.6–12.3%)		8.3% (7.2–9.6%)	
moderate	10.1% (8.3–12.4%)		13.8% (11.8–15.9%)		20.2% (17.9–22.8%)		15.1% (13.4–16.9%)	
worse or bad	31.2% (26.6–36.2%)		34.6% (31.2–38.3%)		42.8% (39.5–46.2%)		37.2% (34.4–40.2%)	
dmft		< 0.001		< 0.001		< 0.001		< 0.001

*P* values are based on chi-square test

Table 4 shows the results of the multivariable logistic regression. In terms of predisposing factors, parents with higher educational levels (OR: 2.29, 95%CI: 1.97–2.67, *p < 0.001*) were more likely to take their children to the dentist than those who had a junior high school or lower education. Abundant oral health knowledge (OR: 1.38, 95%CI: 1.24–1.53, *p < 0.001*) and positive oral health attitudes (OR: 1.64, 95%CI: 1.43–1.89, *p < 0.001*) were associated with a higher probability of visiting the dentist at least once. Regarding enabling factors, children who lived in rural areas were less likely to have visited a dentist in the past 12 months. Children from families who had a high annual per capita income (OR: 1.40, 95%CI: 1.18–1.65, *p < 0.001*) had higher probability of dental visits. With regard to need factors, parents who thought their children’s oral status was “worsening or bad” or considered their children’s overall health as “good” tended to take their children to the dentist. Higher dmft (OR: 1.05, 95%CI: 1.04–1.07, *p < 0.001*) scores were associated with the use of dental care services. In addition, children who had reported a toothache (OR: 9.72, 95%CI: 7.81–12.09, *p < 0.001*) were more likely to visit a dentist than those that had never reported a toothache.

**Table 4:** Logistic regression model of oral health service utilization in the past 12 months

	Model 1		Model 2		Model 3	
	OR (95%CI)	P	OR (95%CI)	P	OR (95%CI)	P
Predisposing factor						
Age						
3	1(reference)		1(reference)		1(reference)	
4	1.38 (1.17–1.62)	< 0.001	1.37 (1.16–1.62)	< 0.001	1.06 (0.89–1.26)	0.50
5	2.19 (1.91–2.52)	< 0.001	2.18 (1.89–2.50)	< 0.001	1.33 (1.15–1.53)	< 0.001
Education						
junior high school or lower	1(reference)		1(reference)		1(reference)	
senior high school	1.44 (1.27–1.63)	< 0.001	1.31 (1.15–1.48)	< 0.001	1.33 (1.15–1.54)	< 0.001
bachelor’s degree or higher	2.39 (2.09–2.75)	< 0.001	1.99 (1.75–2.26)	< 0.001	2.29 (1.97–2.67)	< 0.001
Score of oral health attitude						
0 to 3	1(reference)		1(reference)		1(reference)	
4 to 6	1.71 (1.47–1.98)	< 0.001	1.66 (1.43–1.92)	< 0.001	1.64 (1.43–1.89)	< 0.001
Score of oral health knowledge						
0 to 4	1(reference)		1(reference)		1(reference)	
5 to 8	1.36 (1.24–1.51)	< 0.001	1.33 (1.21–1.46)	< 0.001	1.38 (1.24–1.53)	< 0.001
Enabling factor						
Location						
urban			1(reference)		1(reference)	
rural			0.73 (0.61–0.88)	0.00	0.61 (0.50–0.75)	< 0.001
Region						
west			1(reference)		/	/
middle			0.92 (0.72–1.18)	0.51	/	/
east			1.17 (0.93–1.47)	0.17	/	/
Annual per capital income						
the lowest to 12500CNY			1(reference)		1(reference)	
12500CNY-25000CNY			1.15 (1.00–1.33)	0.05	1.22 (1.05–1.42)	0.01
25000CNY to the highest			1.28 (1.08–1.51)	0.00	1.40 (1.18–1.65)	< 0.001
Need factor						
Toothache						
never					1(reference)	
occasionally					4.49 (3.99–5.05)	< 0.001
often					9.72 (7.81–12.09)	< 0.001
its not known					1.22 (0.92–1.61)	0.17
Evaluation of overall health						
good					1(reference)	
fair					0.81 (0.72–0.92)	0.00
moderate					0.65 (0.57–0.74)	< 0.001
worse or bad					0.56 (0.42–0.75)	< 0.001
Evaluation of oral health						
good					1(reference)	
fair					1.31 (1.11–1.54)	0.00
moderate					1.97 (1.64–2.37)	< 0.001
worse or bad					3.54 (2.84–4.40)	< 0.001
dmft					1.05 (1.04–1.07)	< 0.001

## Discussion

The oral health service utilization prevalence in the past 12 months was 9.5% among 3-year-old children, 12.1% among 4-year-old children, and 17.5% among 5-year-old children. Compared with the Third National Oral Health Survey, among 5-year-old children, the prevalence of utilization of dental care increased slightly, but there is still a gap between utilization in China and the level of dental health care utilization in developed countries. In our study, we aimed to explain the patterns of dental service utilization among 3- to 5-year-old children in China with Andersen’s behavioural model. We find that education, knowledge, attitude, dental pain, evaluated dental health, and dmft scores are associated with oral health utilization, and annual per capita income and rural-urban location can also affect the use of dental service.

Among the predisposing characteristics, demographic factors suggested the possibility of needing health services. In the current study, we found that age had a significant effect on dental care utilization. Oral health service utilization during the past 12 months showed a rising trend with age. This finding could possibly be explained by the cumulative effect of increasing oral problems as children grow [24]. The teeth of older children are exposed to environmental conditions for a longer period of time, which increases the likelihood of dental disease [24, 25]. According to the Fourth National Oral Epidemiology Report [3], the prevalence of dental caries in deciduous teeth increases with age between 3 and 5 years of age. Another explanation is that older children are able to more clearly convey their pain or discomfort to parents and dentists and can better cooperate during dental procedures [20].

For preschool children, parental characteristics play an important role in the utilization of health services. In this study, nearly half of the parents had no more than a junior high school education; regarding reasons for not visiting dentists, approximately 70% of parents believed that their children did not have any dental diseases. This unrealistic optimism may stem from the absence of health knowledge. Oral pain is often mistakenly regarded as the only symptom of perceived disease [26]. Prior studies have also mentioned that in China, traditional Chinese medicine considers the oral cavity to be part of the entire body; therefore, the first treatment modality for dental disease is sometimes home remedies rather than professional treatment [7].

Our results confirmed that family income demonstrated a positive relationship with dental health service utilization. The susceptibility to caries and dental pain is socio-economically and geographically unequal [27]. Children who are disadvantaged by poverty have an increased burden of disease but attend few dental visits [28]. Regarding medical insurance, the reimbursement prevalence for oral diseases is notably low in China [29], and the public must pay out of pocket for clinical treatment. The low-income population is focused on meeting basic needs and is less likely to seek dental care [30]. Furthermore, a prior study confirmed that poverty associated with education leads to reduced knowledge and a poor attitude regarding oral health [31]. Thus, financial barriers have a negative effect on dental care; similar results have been reported in prior studies [32–35].

The results of this study showed that children who lived in urban areas were more likely to receive dental services than those who lived in rural areas. This phenomenon may have occurred because health system-related economic resources are not dispersed homogeneously throughout the country [14]. A shortage in the dental workforce, particularly paediatric dentists, is a common health care issue in low-income areas [36]. The density of pediatric dentists at county (city) level is significantly related to the utilization of oral health care at county (city) level [37]. A similar finding was reported in America, where utilization of dental care showed a negative trend as urbanization decreased [38].

According to Andersen’s behavioural model, perceived illness or the probability of its occurrence was a main reason for seeking dental services. Results from the multiple regression analysis in the present study showed that pain was the strongest factor associated with oral health service utilization. According to prior studies, dental pain, which can lead to difficulty in eating and consequently result in malnutrition and underweight, has become the most important reason for dental treatment [25, 39]. Besides that, our study also showed that parents’ perception of their children’s dental health was an important predictor of a child’s use of oral health care services. Parents who evaluated their child’s dental status as “poor” took their child to the dentist more often than those who evaluated their child’s status as “good”. An explanation for the result might be parents’ beliefs about seeking curative but preventive services [40]. Thus, the utilization patterns of oral health services among preschool children in China are still disease-oriented, and effective methods for promoting preventive dental care need to be expanded. However, parents who considered their child’s overall health to be poor were less likely to bring their child to an oral hospital; this is possibly because whole body health is more urgent than oral health, and thus oral health becomes a secondary concern.

In the present study, the dmft was associated with oral health service utilization. Another study confirms the results [41]. The prevalence of dental caries based on clinical examination was 63.1%, however, only 17.6% of children were reported to have utilized oral health services. This difference suggests that dental need did not translate into demand. A prior study indicated that potential demand is caused by both demanders and suppliers [42]. Due to parents’ lack of awareness or incorrect awareness of oral health, there is no effective demand for treatment of unrecognized diseases. Furthermore, disadvantages associated with medical institutions providing inappropriate services, such as high cost, difficult registration, and long waiting time, among others, are also important factors that affect patients’ visits. A study has shown that oral hygiene service utilization increases slightly, when a child is diagnosed with an oral problem [43]. To achieve the goal of continuously and effectively transforming objective need into subjective demand, the countermeasures that are adopted must be aimed at changing behaviour on both the supply and demand sides. For example, oral examinations may be carried out in school or in the community, and when a child is found to have dental caries, timely treatment may be given.

When calculating the survey weights, data of the 3–5-year-old age group in the Sixth Population Census of China were not available, so we selected the data from 1 to 4-year-old age group as a substitute, which may have affected the accuracy of the results. In addition, per capita household income was obtained by filling in missing values. Although accurate data were utilized to the greatest extent possible, this data substitution may have resulted in deviations to some extent. Furthermore, the present study had some limitations. First, the children in the sample were all from kindergartens, and children not attending kindergartens in remote areas were not included, resulting in selection bias. Second, because the studied factors occurred in the past, recall bias was unavoidable. Third, the questionnaire was completed by the parents, and its accuracy was subject to the parents’ understanding of their children. Fourth, our study is of cross-sectional design, which excludes any inference about causality, A future longitudinal study is highly desirable to address these limitations.

Despite the above limitations, this study was still a comprehensive and systematic reflection of the current utilization of health services by preschool children in China. To improve the oral health of pre-schoolers in China, the study findings have some implications for policy adjustment to increase the utilization of oral health services. First, it is time for the government to pay attention to the inequality in the distribution of dental resources and take measures to solve this problem. Second, expansion of insurance coverage for dental treatments would be beneficial for children who are less able to afford oral health services. Third, promoting parents’ awareness of oral health would increase the prevalence of dental service utilization.

## Conclusion

The present study illustrated that the prevalence of dental service utilization was still relatively low among preschool children. Based on Andersen’s behavioural model, predisposing, enabling, and needs variables as well as oral health practices were associated with increased dental health services utilization. In addition, disparities in oral health care utilization remain and need to be addressed by the joint efforts of the government and the whole society.

## Supplementary information

**Additional file 1:** Oral health assessment form for children.

**Additional file 2:** Oral health questionnaire for parents/caregivers.

## Data Availability

The datasets used during the current study are available from the corresponding author on reasonable request.

## Abbreviations

AAPD: American Academy of Pediatric Dentistry
CPI: Community periodontal index
dmfs: Decayed (non-cavitated or cavitated lesions), missing (due to caries) or filled tooth surfaces
dmft: Decayed, missing and filled teeth
ECC: Early childhood caries
PPS: Probability proportional to size
WHO: World Health Organization
