# Supplementary material for: Utilization of dental services and associated factors among preschool children in China
Source: BMC Oral Health. 2020 Jan 8;20:9. doi: 10.1186/s12903-019-0996-x (PMC6950915; doi:10.1186/s12903-019-0996-x)
Supplement: Supplementary file 1 — Additional file 1: Oral health assessment form for children. [file 12903_2019_996_MOESM1_ESM.pdf]

The 4th national oral health survey（3-5 year）

ID: ____________ Name：____________

Gender：______ Man=1 Female=2 Nation: _____ Census register：____ Urban=1 Rural=2

Birthdate: ________

Year: _______ 2015=1 2016l=2 Date: __________ Inspector number: __________

1．Dentition status

		55	54	53	52	51	61	62	63	64	65	
	16	15	14	13	12	11	21	22	23	24	25	26
Dentition												
Dentition												
	16	15	14	13	12	11	21	22	23	24	25	26
		55	54	53	52	51	61	62	63	64	65	

Primary teeth status	Permanent teeth status	
A	0	Sound
B	1	Caries
C	2	Filled with caries
D	3	Filled, no caries
E	4	Missing due to caries
X	5	Missing for another reason
F	6	Fissure sealant
G	7	Fix prosthesis/crown. abutment, veneer
T	8	Trauma
N	9	Not record

Immediate treatment and treatment scheduled		Table type	
Yes=1		Original=1	
No=2		Review=2	
