# Supplementary material for: Utilization of dental services and associated factors among preschool children in China
Source: BMC Oral Health. 2020 Jan 8;20:9. doi: 10.1186/s12903-019-0996-x (PMC6950915; doi:10.1186/s12903-019-0996-x)
Supplement: Supplementary file 2 — Additional file 2: Oral health questionnaire for parents/caregivers. [file 12903_2019_996_MOESM2_ESM.docx]

**The fourth national oral health epidemiology questionnaire**

**The fourth national oral health questionnaire in 2015 (children's parents)**

**ID: _______________ Name: ____________**

**Date: ____________** **Inspector number: ____________**

Note: only parents and grandparents can complete this questionnaire!

Requirements: please tick "√" in the "□" in front of the corresponding options in multiple choice questions.

1.What is your relationship with the child? **(Choose only one answer)**

1) father 2) mother 3) grandparent

4) relatives 5) babysitter

2. Your child's weight at birth is _ _ _ _ _ _ _ catty. **(Please keep one decimal, fill in "N" if you do not know or refuse to answer)**

3. How does your child eat and drink within six months of birth? **(Choose only one answer)**

1) Entire breast-feeding 2) Primary breast-feeding

3) Entire artificial feeding 4) Primary artificial feeding

5) Half breast-feeding and half artificial feeding

4. How often do your children eat the following foods or beverages? **(Choose one answer for each sub-question.)**

|  | 6 | 5 | 4 | 3 | 2 | 1 |
| --- | --- | --- | --- | --- | --- | --- |
|  | 2 or more times a day | Everyday | 2-6 times a week | Every week | Every month | Little or never |
| 1）Desserts (biscuits, cakes, bread) and candies (chocolate, sugar gum) | ⬜ | ⬜ | ⬜ | ⬜ | ⬜ | ⬜ |
| 2）Sweet beverage (carbonated beverage, juice or non-fresh-pressed juice.) | ⬜ | ⬜ | ⬜ | ⬜ | ⬜ | ⬜ |
| 3）Sweetened milk, yogurt, milk powder, tea, soy milk, coffee | ⬜ | ⬜ | ⬜ | ⬜ | ⬜ | ⬜ |

5.Do your child eat desserts or drink sweet drink before sleep? **(Choose only one answer)**

1) often 2) occasionally

3) never

6. Do your child brush teeth? **(Choose only one answer)**

1) Yes 2) seldom or never **(if you choose this answer then turn to question No.7-11 directly)**

7. When do your child start brush teeth? **(Choose only one answer)**

1) 6 months after birth 2) 1 year old 3) 2 years old

4) 3 years old 5)4 years old 6) 5 years old

7) I don't remember

8. How many times does your child brush his teeth every day? **(Choose only one answer)**

1) 2 times and above 2) 1 time 3) not every day

9. How often do you help your child brush his/her teeth? **(Choose only one answer)**

1) everyday 2) every week 3) sometimes 4) occasionally 5) never

10. Does your child use toothpaste when brushing his teeth? **(Choose only one answer)**

1) Yes 2) No

3) I don’t know (If you choose 2 or 3, you don’t need to answer Question 11)

11. Does your child use fluoride toothpaste when brushing his teeth? **(Choose only one answer)**

1) Yes 2) No 3) unknown

12. How often did your child have any toothache or discomfort in the past 12 months? **(Choose only one answer)**

1) never 2) occasionally 3) often 4) I don’t know

13. Did your child have any dental visit? **(Choose only one answer)**

1) Yes 2) Never **(choose 2 not to answer questions 14 to 17)**

14.When was the last dental visit from now? **(Choose only one answer)**

1) less than 6 months 2) 6-12 months **(choose 1or2 not to answer questions 18)**

3) more than 12 months **(choose 3 not to answer questions 15 to 17)**

15. What was the reason for your child’s last dental visit? **(Choose only one answer)**

1) consulting and check out 2) preventive dental care

3) receiving treatment 4) unknown

16. How much did you spend on your child’s dental visit?

Please write down here: _____yuan. **(Please fill in an integer. If you do not know or refuse to answer, fill in "N")**

17. Among the above-mentioned dental expenses, the proportion you personally need to pay is _ _ _ _ _ _%. **(Please fill in an integer. If you do not know or refuse to answer, fill in "N")**

18. Why didn't your child go to the hospital for a dental visit in the past 12 months? **(You can choose more than one answer)**

1)No dental diseases 2) Dental disease was not severe

3)No need to cure primary teeth 4) Economic issue

5)Inconvenience 6) No time

7)Fear of pain 8) No dentists nearby

9)Fear of infectious diseases 10) No reliable dentists

11)Difficulty of registration 12) Seeing dentists in kindergarten

13)Other reasons

19. How do you evaluate the overall health of your child? **(Choose only one answer)**

1)good 2) fair 3) moderate 4) bad 5) worse

20.How do you evaluate the oral health of your child? **(Choose only one answer)**

1)good 2) fair 3) moderate 4) bad 5) worse

21. How do you think of the following points of view? **(Choose only one answer for every sub-question)**

|  | 1 | 2 | 8 | 9 |
| --- | --- | --- | --- | --- |
|  | agree | disagree | do not care | unknown |
| 1)Oral health is very important to our life. | ⬜ | ⬜ | ⬜ | ⬜ |
| 2)Regular oral examination is essential | ⬜ | ⬜ | ⬜ | ⬜ |
| 3)Our teeth are instinctive and there is little to do with self-protection. | ⬜ | ⬜ | ⬜ | ⬜ |
| 4)To prevent tooth disease, first of all, by yourself | ⬜ | ⬜ | ⬜ | ⬜ |
| 5)It's important to protect your child's six-year-old teeth | ⬜ | ⬜ | ⬜ | ⬜ |
| 6)Poor conditions of the mother’s dental health would affect the children’s dental health condition | ⬜ | ⬜ | ⬜ | ⬜ |

22. How do you think of the following statements? (Choose only one answer for every sub-question)

|  | 1 | 2 | 8 |
| --- | --- | --- | --- |
|  | correct | incorrect | I don’t know |
| 1)Gingival bleeding during brushing is normal. | ⬜ | ⬜ | ⬜ |
| 2)Bacteria can cause gingivitis. | ⬜ | ⬜ | ⬜ |
| 3)Brushing your teeth is no use in preventing gum bleeding. | ⬜ | ⬜ | ⬜ |
| 4)Bacteria can cause dental caries. | ⬜ | ⬜ | ⬜ |
| 5)Sugar can cause dental caries. | ⬜ | ⬜ | ⬜ |
| 6)If primary tooth is diseased, it doesn’t need to be treated | ⬜ | ⬜ | ⬜ |
| 7)Pit and fissure sealant can prevent children from dental caries | ⬜ | ⬜ | ⬜ |
| 8)Fluoride can’t protect teeth | ⬜ | ⬜ | ⬜ |

23. What is your highest educational degree? **(Choose only one answer)**

1) illiteracy 2) primary school 3) junior high school

4) senior high school 5) technical secondary school 6) junior college

7) bachelor’s degree 8) master’s degree and above

24. How many people are there in your family? _____ **(If you don't know or refuse to answer, please write "N".)**

25. What was the total income of the person in your family in the last 12 months? _____*10000 RMB /year **(please fill in an integer number, or "N" if you don't know or refuse to answer)**

**Thank you for your cooperation!**
